# Staging and defect-limited intercalation of FeCl_3_ in graphite electrodes

**DOI:** 10.1038/s41467-026-74399-w

**Published:** 2026-06-16

**Authors:** Peter Schweizer, Lilian M. Vogl, Colin Ophus, Andrew M. Minor

**Affiliations:** 1 National Center for Electron Microscopy (NCEM), Lawrence Berkeley National Laboratory, Berkeley, CA USA; 2 Department of Materials Science and Engineering, University of California Berkeley, Berkeley, CA USA; 3 Department of Materials Science and Engineering, Stanford University, Stanford, CA USA

**Keywords:** Batteries, Two-dimensional materials, Transmission electron microscopy

## Abstract

The need for sustainable mobility and renewable energy systems has been a major driving force for battery research in recent years. While ion batteries are in widespread application, there remain unsolved questions regarding fundamental processes occurring within batteries during use that ultimately limit their performance. The most important of such processes is intercalation, which describes the reversible incorporation of a guest species into a host lattice. The early stages of this process, when only a limited amount of guest material is present, are still barely understood. In this work, we use advanced transmission electron microscopy to directly observe the structure and host/guest interactions in a partially intercalated graphite model system. We show the three-dimensional layer occupancy and demonstrate that the established staging laws break down in this regime. Finally, we elucidate the impact of host lattice defects on the intercalation process using 4D-STEM, moiré imaging and in situ heating.

## Abstract (web summary)

Using transmission electron microscopy, this study reveals the 3D structure of partially intercalated graphite, showing breakdowns in conventional staging behavior and highlighting how lattice defects govern early-stage intercalation.

## Introduction

Batteries and battery materials have been at the forefront of scientific research in recent years since they play an integral role in enabling sustainable technologies^1–5^. In particular, electrode materials for ion batteries have been studied extensively using a plethora of different methods^6–8^ in a quest for improved capacity^9–12^, stability^13–15^, and lifetime^16,17^. While conversion-type electrodes demonstrate promising properties such as very high capacities, they typically suffer from lower stability and unresolved safety concerns^18–22^. Most current ion batteries in application instead make use of intercalation/insertion-based electrodes that show high cyclic stability and low hysteresis^23^. Energy storage in these systems relies on the reversible and repeatable insertion of a guest species (i.e., ions) into a host lattice (the electrode material). This process, called intercalation, is highly complex and to this day not yet fully understood. For a layered host material such as graphite, occupancy of interlayer gaps during intercalation is described by so-called staging models. These models, like the Rüdorff-Hoffmann^24^ and Daumas-Hérold^25^ models, describe which layers are occupied at a certain degree of intercalation (see Supplementary Fig. 1 for schematics of the models). However, these models are not applicable to dilute intercalation as it would be found in battery electrodes at very low battery charge (or both high and low charge if both electrodes are layered). Understanding what mechanisms govern early-stage intercalation is expected to increase the lifetime and cyclability of ion batteries. One sizeable challenge in analyzing sparsely intercalated electrodes is the requirement to detect small fractions of one material inside the bulk of another material. For layered materials, this translates to detecting atomic monolayers inside a hundred to a thousand times thicker host lattice. While bilayer graphene or graphene/buffer layer host systems can be used to study intercalation dynamics and host/guest interactions at the atomic scale^26–28^ they cannot represent real-world materials which are orders of magnitude thicker and have a complex three dimensional interplay of host and guest system. Therefore, in this work, we use exfoliated Bernal-type graphite flakes with a thickness of >100 layers to be able to study: (i) the three-dimensional distribution of guest species at the early stages of intercalation and (ii) to analyze the effect of defects on intercalation dynamics. This analysis is enabled by advanced transmission electron microscopy (TEM) techniques such as four-dimensional scanning transmission electron microscopy (4D-STEM) and in situ heating.

## Results

**Fig. 1:**
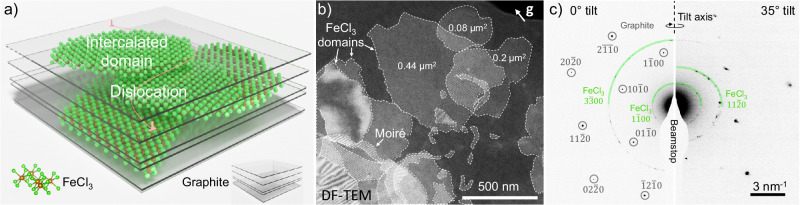
Structure of a partially intercalated FeCl_3_/Graphite compound. **a** Structural model of the compound: domains of crystalline FeCl_3_ are sandwiched between graphite sheets. Structural defects such as dislocations are ubiquitous in the host lattice. **b** Overview dark-field TEM image of the compound material showing partially intercalated layers of varying size. The objective aperture was placed around FeCl_3_ $3\bar{3}00$ reflections as indicated and shown in Supplementary Fig. 3. Overlapping of layers on different van-der-Waals gaps leads to the formation of Moiré patterns. **c** Electron diffraction patterns of the intercalation compound at 0 and 35° tilt showing a rotational degree of freedom and 2D structure of the intercalated material. Simulated diffraction patterns and details regarding the diffraction tilt series are shown in Supplementary Fig. 5 and Supplementary Movie 1.

Here we study an Iron(III)-chloride/graphite intercalation compound, which is a promising material for battery electrodes in and of itself ^29–31^ but which also serves as a model system to study host/guest interactions in intercalated graphite in general. Figure 1 a shows the structural model of the sample system schematically. At the early stage of intercalation, FeCl_3_ occupies only a few of the graphite interlayer gaps. The guest species forms incommensurate two-dimensional crystalline domains inside of graphite’s van der Waals gaps^32,33^. Graphite in its natural state hosts many defects, with basal plane dislocations being especially abundant^34,35^ and it can be expected that these defects interact with intercalated layers^36^. To study the structure of the intercalation compound, we use transmission electron microscopy (TEM), which provides the necessary spatial resolution and sensitivity to single intercalated layers. The fact that the intercalated species forms crystalline domains can be exploited in TEM by using specific diffracted beams to form dark-field (DF) images that only show the intercalated species and block out the contribution of the host lattice (see Supplementary Fig. 2 for details on image formation). This enables us to be sensitive enough to see FeCl_3_ monolayers inside a > 100 times thicker host lattice. Figure 1b shows an exemplary TEM dark-field image of FeCl_3_ intercalated in graphite (see Supplementary Fig. 3 for the corresponding diffraction pattern and aperture placement). Rather than full layers being occupied, we observe distinct independent domains of the intercalant (outlined with white dashed lines). Energy dispersive X-ray spectroscopy confirms that the domains originate from iron chloride (see Supplementary Fig. 4). Some of these domains are overlapping, indicating that they are populating different van-der-Waals gaps of the host material. We also observe Moiré patterns in some of the overlapping regions, which originate from rotational misorientations and strain. The moiré patterns are also sensitive to defects within intercalated layers. Intercalated domains are evenly dispersed over the observable area of the graphite flakes. Looking at a selected area diffraction (SAED) pattern (see Fig. 1c left side), we see complete rings for FeCl_3_ compared to distinct spots for graphite. This indicates a rotational degree of freedom for the intercalated species within the single-crystalline host lattice. Furthermore, the presence of the normally forbidden $1\bar{1}00$ ring confirms the quasi-2D nature of the intercalated layers. Tilting the specimen 35° relative to the graphite basal planes (Fig. 1c right side) leads to the emergence of an elliptical shape for this diffraction ring (stretched horizontally), which is a further indication for the 2D nature of the intercalant material (simulated diffraction patterns for 3D and 2D FeCl_3_ as well as more details on the diffraction tilt series are found in Supplementary Fig. 5). The preparation process is highly reproducible, with different samples showing the same type of intercalated structures (see Supplementary Fig. 6 for more details on different samples). At the early stages of intercalation, the intercalated material is concentrated inside a few different graphite van der Waals gaps, where it forms continuous domains of varying size. The area of these domains varies between 10^−3^ µm^2^ and 2 µm^2^ with a seemingly arbitrary distribution and partial overlap in some regions but not in others. The outline of certain domains shows a small degree of faceting, which in some cases is aligned with lattice planes in the graphite host lattice (see Supplementary Fig. 7).

### 3D distribution of intercalated material

**Fig. 2:**
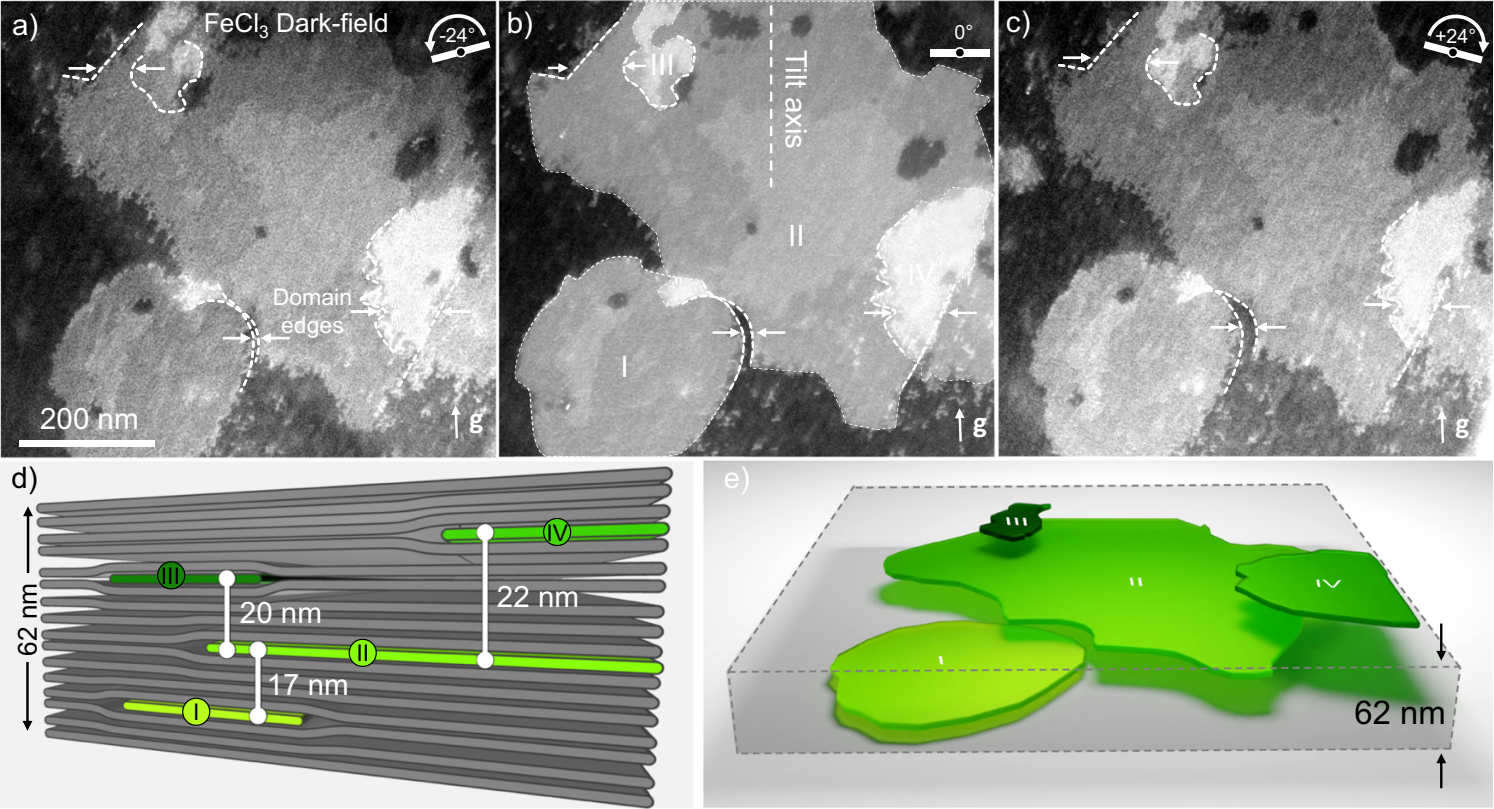
3D structure of partially intercalated graphite. **a–c** Dark-field TEM images at −*24*, 0 and +24 ° tilt angles, showing parallax between different layers (numbered I-IV) occupying different van-der-Waals gaps. The g-vector was chosen along the tilt axis as indicated. Full tilt series in Supplementary Movie 2. The white arrows indicate the projected distance between the edges of different intercalated layer edges (outline by dashed lines) from which the vertical separation is calculated. From the changing distance **d** Schematic representation of the calculated distance between intercalated layers inside of the graphite stack which has a thickness of around 62 nm (only about 10% of graphite layers are actually drawn). **e** 3D rendering of the reconstructed volume of FeCl_3_ intercalated graphite. Intercalated material is found in larger continuous domains on different layers, fairly spread out in the vertical direction.

Besides the lateral extent of the intercalated domains, we also studied the vertical distribution of these layers within the host lattice using dark-field electron tomography^37^. By acquiring images of FeCl_3_ layers at different tilt angles, we can reconstruct their position in a 3D volume, as shown in Fig. 2. Using this analysis, we can distinguish 4 distinct layers (I to IV) that partially overlap at 0° tilt (see Fig. 2b). Upon tilting, the distance between the edges of the different layers and the overlap area changes. For instance, the distance between the marked edges of layers I and II decreases when tilting counter-clockwise (Fig. 2a) but increases when tilting clockwise (Fig. 2c). Due to the 2D nature of the FeCl_3_ layers, their intensity in the DF-images changes little during tilting. With the tilt angle and the change in lateral layer distance, we can calculate the vertical separation of the intercalated domains (see Supplementary Fig. 8 for details). Figure 2d shows schematically the distribution of the different intercalated layers inside of the graphite host lattice. In this particular case, the smallest distance between layers (III to IV) is around 2 nm (equaling around 6 graphite layers) and the largest distance (I to IV) is 39 nm (equaling around 115 graphite layers). The thickness of the graphite flake can be approximated by the position of surface contamination to around 62 nm, which is equal to 185 graphite layers. With the assumption that the intercalated layers are largely flat, we can segment the lateral extent of the layers and approximate the three-dimensional volume of the entire structure (shown in Fig. 2e). There are a few observations that can be made about the distribution of intercalated material: The iron chloride intercalant agglomerates into continuous layers across different van der Waals gaps, vertically distributed within the graphite. While it could be expected that the intercalated material would form circular domains with a minimized surface, this is surprisingly not the case. Instead, complex shorelines are visible with both concave and convex features, pointing towards some interaction with the host lattice that makes these shapes more favorable.

### Interactions between graphite defects and intercalated layers

**Fig. 3:**
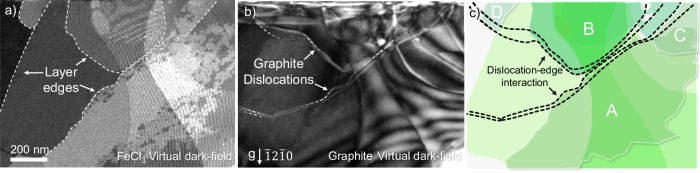
Host/guest interactions in graphite/FeCl_3_ intercalation compound analyzed with 4D-STEM. **a** Reconstructed virtual dark-field image of all intercalated domains inside of a graphite flake. Moiré patterns emerge due to a rotational misalignment between different layers. **b** Virtual dark-field image of graphite showing several basal plane dislocations (indicated with dashed lines). **c** Overlay of basal plane dislocations with intercalated layers showing a clear correlation between the defect lines and layer edges. The domains that show the strongest interaction have been labeled A-D. Mean 4D-STEM diffraction pattern and virtual aperture positions shown in Supplementary Fig. 9. All images are from the same region and share a scale bar.

It is clear that the distribution and shape of intercalated domains are influenced by the host lattice. Because the graphite tested in this work is single crystalline (see diffraction pattern in Fig. 1), we can rule out the effects of grain boundaries and instead can focus on the impact of dislocations. These topological line defects (sometimes also called domain walls or solitons) are ubiquitous in graphite and its derivatives^34,35,38^. In order to characterize the impact of dislocations, we acquired four-dimensional scanning transmission electron microscopy (4D-STEM) datasets that consist of nanobeam diffraction patterns at each scan point of a scan array^39^. This allows us to reconstruct the structure of the host and guest lattice in the intercalation system at the same time and look for correlations between the two. Figure 3a shows a reconstructed dark-field image of intercalated domains in a graphite flake. This image is analogous to the previously shown dark-field images, but 4D-STEM allows us to apply virtual aperture shapes that enable visualization of all of the intercalated domains together and irrespective of in-plane rotation. Like the samples shown in Figs. 1 and 2, we observe an arrangement of intercalated domains occupying different van-der-Waals gaps and partially overlapping with each other. The rotation angle between layers varies from 1° to 30°, leading to different Moiré patterns. The 4D-STEM scan also contains diffracted beams of graphite, which can be used to reconstruct virtual dark-field images of the host lattice (as shown in Fig. 3b). The DF image shows distinct bright lines, which can be attributed to basal plane dislocations. Depending on the virtual dark field condition, some of the dislocations become invisible, which can be utilized to determine the direction of their Burgers vector according to the g.b = 0 criterion. The virtual dark-field image in Fig. 3b has been reconstructed from the indicated $(\bar{1}2\bar{1}0)$ reflection, which means that all dislocations that are invisible in that image have a Burgers vector perpendicular to that reflection. If we assume that the dislocations are located on the basal plane, this leads to an effective Burgers vector along the $\left[10\bar{1}0\right]$ direction. This means that the observed dislocations are partial dislocations, with a Burgers vector of magnitude of $\pm \frac{a}{3}\,$ which is expected for graphite^34^. By combining the information obtained from different virtual dark-field conditions, the full structure of the intercalated and host material can be analyzed with knowledge of the Burgers vectors of the basal plane dislocations (see Supplementary Fig. 9 for full details on the Burgers vector determination). Overlaying the identified dislocations onto the intercalated layers (see Fig. 3c), it is immediately apparent that there is a correlation between the intercalant layer edges and the dislocation lines at several points. In particular, the outline of layers A & B correlates strongly with dislocation lines. Some of the lines appear to correlate more strongly with layer edges than others. This may be linked to the Burgers vector and line orientation, which determine whether a dislocation is predominantly edge or screw type. In this case, edge dislocations seem to be more strongly interacting with intercalated layers compared to screw-type dislocations. This direct correlation between dislocations in the host and intercalated layers confirms the significant impact of the defects in the host lattice on the guest species.

### In situ observation of domain growth

**Fig. 4:**
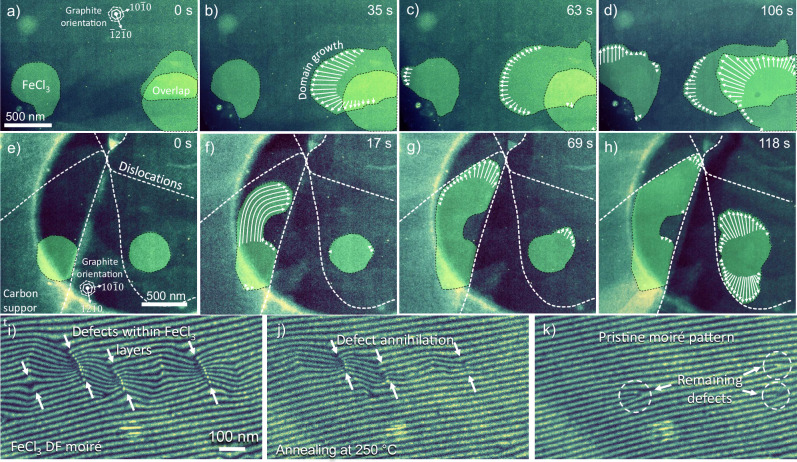
In situ observation of thermally stimulated layer growth and defect interactions. **a**–**d** Case of FeCl_3_ domain growth while cooling from 350 °C to 220 °C over a time period of 106 seconds. Existing layers grow by the attachment of more material. Overlap of layers does not inhibit the further growth of them. The graphite lattice orientation is shown for reference. **e–h** Growth of intercalated domains in the presence of graphite dislocations (marked as white dashed lines). The spatial extent of intercalated domains is limited by the dislocation lines. Full videos are shown in Supplementary Movies 4 and 5. **i–k** Defects within FeCl_3_ layers observed using DF moiré imaging (marked with white arrows). Some defects disappear during heating, while others (outlined with dashed circles) remain. All images are false colored DF-TEM images, raw images are provided as source data.

In order to visualize the dynamic interactions that occur between the host and guest system during intercalation, we employed in situ TEM heating up to 350 °C. At this temperature, we can expect a significant vapor pressure of FeCl_3_^40^, which stimulated a redistribution of intercalated material. Upon heating above 250° intercalated domains start to shrink and change their shape (see Supplementary Fig. 10 and Supplementary Movie 3). Until the chosen setpoint of 350 °C, the crystalline layers become smaller and smaller, with only some of the layers remaining at the end. The shrinkage of intercalated domains suggests that FeCl_3_ molecules detach from the crystalline regions and freely diffuse inside the graphite van-der-Waals gap. During cooling, the free FeCl_3_ molecules start to crystallize again, attaching to remaining domains and/or forming new ones. Overall, no significant loss of intercalant material was found during the procedure, leaving us to believe that the FeCl_3_ mostly remains within the host lattice during heating. Ex situ and beam-off experiments confirm that the observed transformations are thermally stimulated and not an artifact of e-beam illumination (see Supplementary Fig. 11). Figure 4a–d show dark-field TEM images during the cooling part of the in situ experiments. At 350 °C there are three leftover domains present in the sample area. During cooling for 35 s one of the layers starts to grow (extent of growth marked by white arrows in Fig. 4b), which indicates that material diffuses to it and crystallizes. After further cooling (for 106 s), the other layers present in the field of view also increase in size. After cooling to below 220 °C, no significant further growth could be observed. Interestingly, the overlap of layers does not seem to influence the growth of layers. The growth of the domains occurs in waves with fast growth followed by a short period of stagnation. In these growth intervals, we see typical growth rates between 10^−2^ µm^2^ s^−1^ and 0.2 µm^2^ s^−1^. Overall, the growth follows a logistics curve, suggesting that during the periods of intense growth, there is a vast supply of FeCl_3_ molecules present. There is a weak correlation between the orientation of the graphite host lattice and the intercalated layers as inferred from the formation of facets following crystallographic planes of graphite (see Supplementary Fig. 12). In a second case (shown in Fig. 4e–h), we examined a sample area where graphite basal plane dislocations are present (marked as dashed lines). Like in the previous case, there is significant growth of preexisting intercalated domains during cooling. In this case, however, the spatial extent of the layers is clearly influenced by defects in graphite. The domain on the left-hand side is limited by two dislocations, which lead to the formation of an elongated shape of the domain after cooling. A concave shape develops in the outline of the intercalated material, which stays constant during the process. A second layer on the right-hand side of the field of view starts to grow at the later stages of cooling. Its extent is also limited by another graphite dislocation to the left of it. Overall, the process of layer growth seems to be strongly influenced by the defects in the host lattice and to a lesser extent by the orientation of the graphite lattice. We did not observe a phase transformation of FeCl_3_ to FeCl_2_ during heating, as it has been published by Liu et al.^41^ for the case of bilayer graphene and high-resolution imaging. We attribute this to the comparatively lower dose conditions and the thicker host lattice. In addition to observing interactions with the graphite host lattice, we were also able to analyze the evolution of defects within the FeCl_3_ layers by looking at moiré patterns generated by overlapping layers. In the as-intercalated case, we observe many defects within the layers (see Fig. 4i). Upon heating, some of these defects are annihilated by local reorganization of intercalated species (Fig. 4j), while other defects are stable and remain within the layers.

## Discussion

**Fig. 5:**
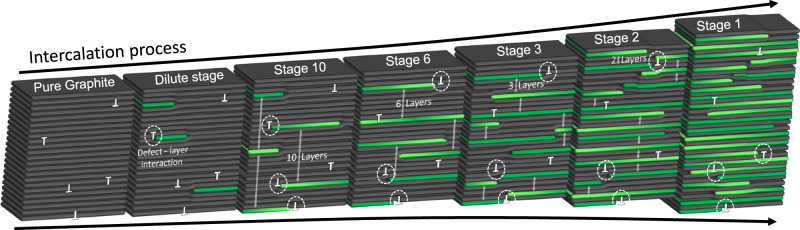
Schematic of a stochastic simultaneous intercalation model based on our results. The host material contains pre-existing defects in the form of dislocations. During initial intercalation, occupied layers are chosen at random. Further insertion of new material proceeds on layers so that occupied layers are equidistant. Defects limit the extent of intercalation on individual layers, leading to partial occupancy. This allows for staging to proceed from the dilute stage all the way to stage 1.

The combined 4D-STEM and in situ heating methods provide a window into the nature of embedded molecular monolayers inside a significantly thicker host lattice. Here, we used the combination of FeCl_3_/Graphite to understand the distribution of intercalated material in the dilute stages of intercalation. The Rüdorff-Hoffmann model suggests that during intercalation, an entire interlayer gap of the host material should be filled with a guest species before intercalation starts on the next one. This model has always suffered from the problem of improbable transitions between different stages. Therefore, the Daumas-Hérold model was developed, which suggests that intercalated material is present equally on all layers throughout the process. However, neither model accurately represents the distribution we find for a dilute stage of intercalation. Instead, material is concentrated on a few domains with complex shorelines that are distributed throughout the volume of the host material. Interestingly, the domains can be located far inside the graphite flakes, instead of being located directly at the edges. This mirrors early work on graphite intercalation in which isolated islands inside larger graphite pieces were found as well^36,42^. The distribution makes sense for a real intercalation process, in which insertion of material may start at different locations on the sample at the same time. Even from this seemingly disordered starting point, lower stages up to complete occupancy can be reached as demonstrated in our stochastic simultaneous intercalation model shown in Fig. 5. Since no layer is fully occupied, it is possible to insert new material in such a way that occupied layers are spaced out in an equidistant fashion. This model can be seen as an extension of the Dumas- Hérold model which also predicts islands on separate layers but does not include local effects such as defects and the complex shapes of the islands.

The lateral extent of intercalated domains is at least in part restricted by defects in the host lattice. While the effect of defects on intercalation has been suggested before, the 4D-STEM analysis and in situ experiments now directly demonstrate the interaction between basal plane dislocations and intercalated domains. This interaction intuitively makes sense as intercalated domains can be viewed as dislocation loops with a non-lattice Burgers vector perpendicular to the basal planes of graphite,^32^ similar to a prismatic edge dislocation^43^. The strain fields of dislocations interact, leading to either an attractive or repulsive interaction depending on the exact configuration and Burgers vectors of the defects. In the case of intercalated layers and graphite dislocations, there is generally an attractive force if the basal plane dislocations are on planes close to one that contains intercalated material (see Supplementary Fig. 13 for calculated stress fields of dislocations^44^ and intercalated layers). This interaction leads to a strong correlation between the extent of intercalated domains and the defect structure of the host lattice. Dislocations are ubiquitous in graphite and typically spread out through the volume. This makes it very likely that dislocation/intercalated domain interactions occur for any given graphite flake. Similar interactions have been observed in the sulphuric acid/graphite system^45^ and for electrochemical intercalation of Lithium in a graphene/Silicon carbide buffer layer^27^. In addition to interactions with host lattice defects, we also see defects within the intercalated layers themselves, which are present upon initial intercalation. In situ heating and cooling reveal that defects can significantly constrain domain growth within specific interlayer gaps. In battery electrodes, this effect could cause incomplete intercalation/deintercalation and material trapping in the host lattice. Trapped material lowers the usable capacity of the battery and therefore may degrade the performance and cycling stability. Defects within the intercalated material are formed as well, which also have a small potential effect on the theoretical battery capacity. We observe nucleation of new domains far inside of graphite flakes instead of directly at the edges, which means that after insertion, the guest species can diffuse around before forming coherent crystalline areas. While our results are first and foremost applicable to incommensurate intercalants like FeCl_3_ and vapor phase intercalation, we believe that many of the observed effects will be similar for other intercalants and may be transferable to electrochemical intercalation under certain circumstances. For the case of lithium, it is conceivable that interactions between host lattice dislocations and the intercalated species occur in a similar way, especially since Li intercalation introduces an additional basal plane shift leading to the formation of stacking faults. However, this remains speculative, and it may as well be the case that Li ions behave differently from the considerably larger FeCl_3_. It would therefore be of significant interest to perform similar experiments on Li intercalation compounds. While other anode materials with a potentially higher capacity are being researched, graphite remains the material of choice, which is why it is important to look into how it can be improved.

In summary, we have analyzed the three-dimensional distribution of intercalated material in a FeCl_3_/Graphite intercalation compound and demonstrated a method to image both the intercalant and host simultaneously with their crystalline defects. We find a pseudo-random distribution of intercalant layers not following the traditional staging laws at the dilute stage. We show that the lateral extent of intercalated layers is strongly influenced by defects in the host lattice. Finally, using in situ heating to mimic the intercalation process, we observed the dynamic redistribution of FeCl_3_ within graphite and found a strong influence of dislocations on the process, with edge dislocations having a greater effect than screw dislocations. We believe that building on the current work, similar results can be obtained for other graphite intercalation compounds as well.

## Methods

### Sample preparation

Natural graphite flakes have been bought from Naturgraphit GmbH (Germany). These graphite flakes have been exfoliated using the scotch tape method and transferred to silicon wafer pieces. Suitable flakes with a thickness between 20 nm and 200 nm have been selected and transferred to TEM grids in the following way: Quantifoil (Quantifoil Micro Tools, Germany) grids have been placed film side down onto the graphite flakes. A drop of isopropanol is put onto the grid, and the sample is put on a hot plate at 200 °C for 5 min. Then the wafer piece with the TEM grid is put into 5% KOH. After some time, the grid separates from the silicon, with the graphite flakes being transferred. The grids are subsequently rinsed multiple times in DI water and Isopropanol. The so-prepared TEM grids with graphite flakes are put in a glass vial together with Iron Chloride powder. The vial is sealed and heated to 250 °C for 5 min which leads to intercalation. After the intercalation process, the samples are rinsed in deionized water to remove excess FeCl_3_ powder for the final product. To make sure that the rinsing process does not influence the structure of intercalated domains, selected samples were investigated without this step. The domain structure is the same for both sample types (see Supplementary Fig. 14).

### Electron microscopy

All transmission electron microscopy has been performed at 80 keV to avoid knock-on damage. Dark-field images and diffraction patterns have been recorded on TitanX and Titan Themis *(*Thermo *Fisher Scientific, USA)* instruments outfitted with Ultrascan and Ceta Cameras, respectively. Typical acquisition times for dark-field images were in the range of 2 s to 30 s for still images and between 0.79 and 1.43 s for in situ movies. In the in situ videos, the dose rate was around 12 e^−^A^−^2s^−1^; for the 4D-STEM scans, the dose rate equaled approximately 0.018 e^−^A^−^2s^−1^.

4D-STEM scans were acquired at a Titan Themis (Thermo Fisher Scientific, USA) using a K2 camera Gatan, USA) in summit mode. 4D-STEM parameters for Fig. 3: 250 × 200 pixels scan size, 7.5 nm step size, dwell time 100 ms. In situ heating was performed with a Gatan 652 furnace-type heating holder with both Titan Themis and TEAM I microscopes (using a Ceta camera in the former and a Gatan K3 Camera in the latter).

### Data evaluation

The evaluation of 4D-STEM datasets has been performed using the py4DSTEM^46^ package. Virtual dark-field images have been reconstructed using virtual apertures put around corresponding reflections. The images of the in situ videos have been aligned using a cross-correlation method.

## Supplementary information

Supplementary Information

Description of Additional Supplementary Files

Supplementary Movie 1

Supplementary Movie 2

Supplementary Movie 3

Supplementary Movie 4

Supplementary Movie 5

Transparent Peer Review file

## Source data

Source Data

## Data Availability

Source data are provided with this paper. Further data supporting the key findings of this study are available within the supplementary Information file and supplementary movies. Raw data generated in this study have been deposited in the Zenodo database (10.5281/zenodo.20111771).Source data are provided with this paper.
