# Supplementary material for: Staging and defect-limited intercalation of FeCl3 in graphite electrodes
Source: Nat Commun. 2026 Jun 16;17:7609. doi: 10.1038/s41467-026-74399-w (PMC13424089; doi:10.1038/s41467-026-74399-w)
Supplement: Supplementary file 1 — Supplementary Information [file 41467_2026_74399_MOESM1_ESM.pdf]

Staging and Defect Limited Intercalation of $FeCl_3$ in Graphite Electrodes

Peter Schweizer[1], Lilian M Vogl[1,2], Colin Ophus[3], Andrew M Minor[1,2]

[1] National Center for Electron Microscopy (NCEM), Lawrence Berkeley National Laboratory, Berkeley, CA, USA

[2] Department of Materials Science and Engineering, University of California Berkeley, Berkeley CA, USA

[3]Department of Materials Science and Engineering, Stanford University, Stanford, CA, USA

Supplementary Information

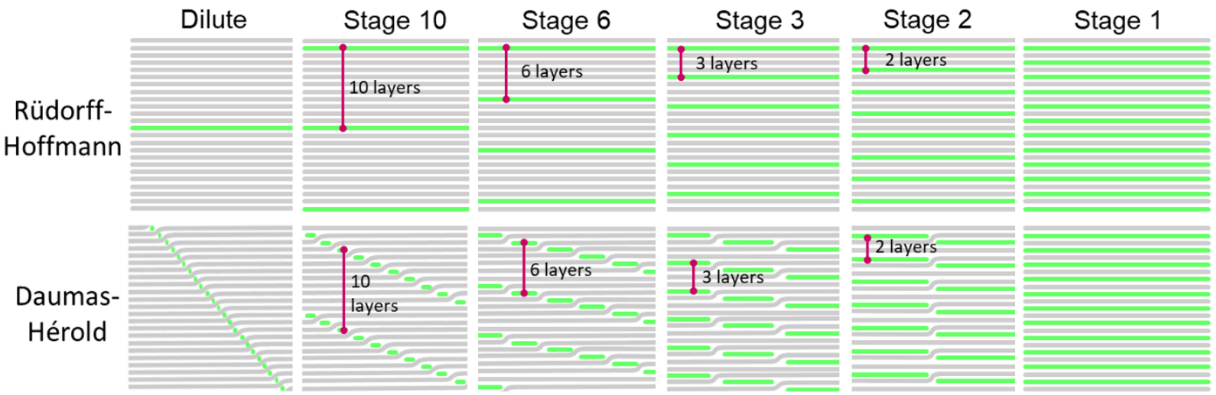

Supplementary Figure 1: Schematic of established staging models. In the Rüdorff-Hoffmann model, entire interlayer gaps of the host lattice (shown in grey) are filled one by one with the guest species (shown in green) during intercalation. The distance between filled layers generally is the same throughout the material. This model suffers from the fact that there is no plausible transition between certain stages during the intercalation process. In contrast the Daumas-Hérold model suggests that intercalation happens on all interlayer gaps at the same time. This makes stage transitions possible while also keeping a constant distance between filled layers. Defects are not accounted for in both models.

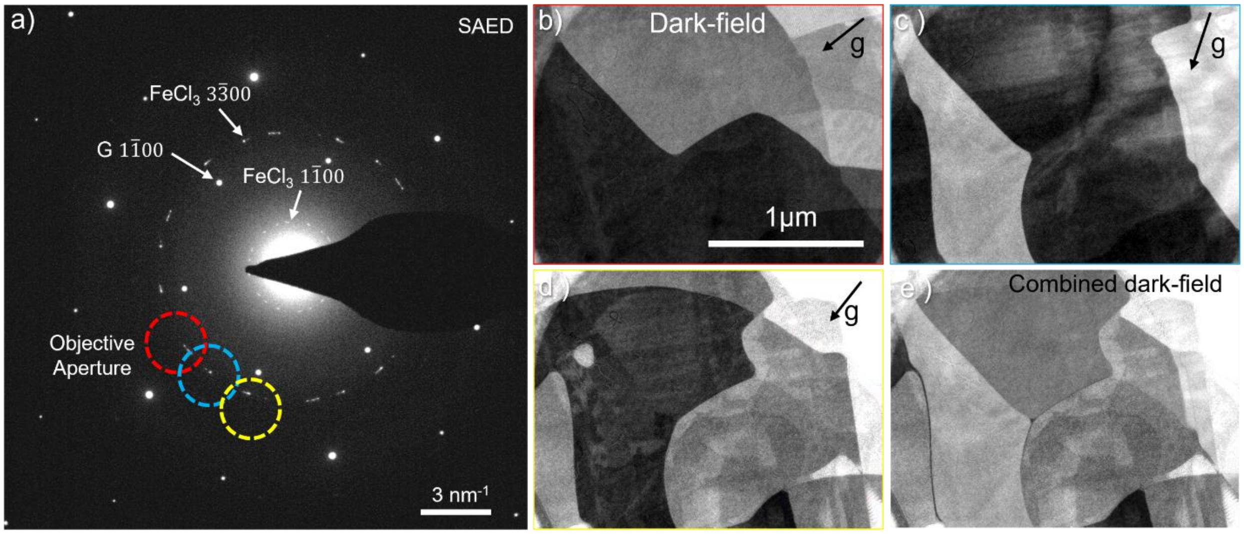

Supplementary Figure 2: Dark-field image formation for intercalated layers. a) Exemplary selected area electron diffraction pattern showing single crystalline graphite and polycrystalline iron chloride spots. By placing an objective aperture around the iron chloride spots and avoiding graphite reflections, images of only the guest species can be acquired. Exemplary aperture positions are shown in red, blue and yellow with corresponding dark-field images shown in b), c) and d). Combining different dark-field images gives access to the complete structure of the occupied layers.

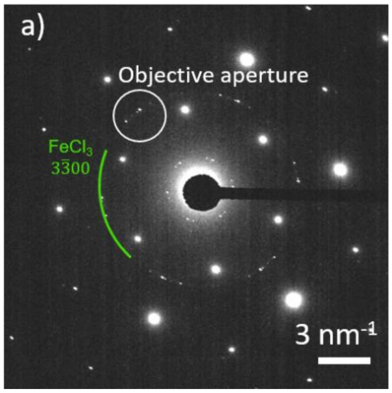

Supplementary Figure 3: Diffraction pattern and aperture position corresponding to figure 1b.

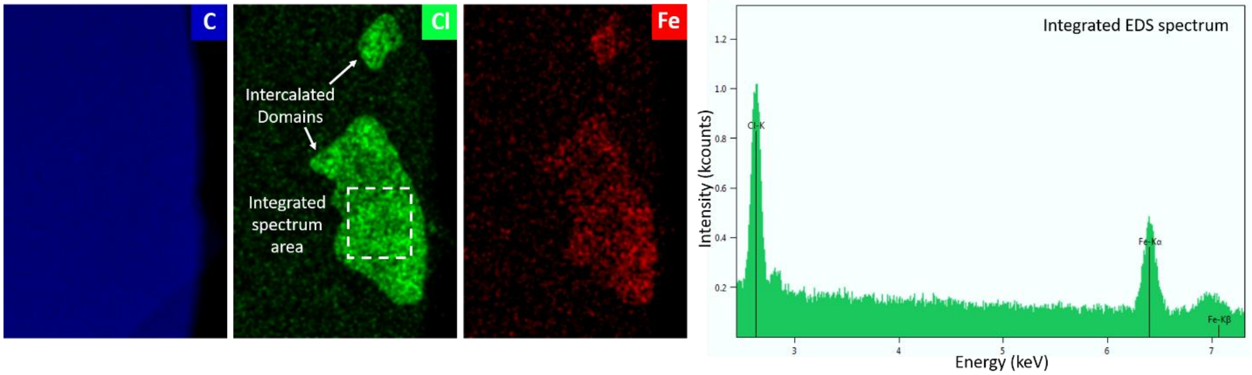

Supplementary Figure 4: Energy dispersive x-ray spectroscopy mapping of intercalated layers. Elemental maps of carbon, chlorine and iron are shown side-by-side. Two intercalated domains containing both iron and chlorine are present in the field of view. A sum spectrum integrated over one of the intercalated domains shows chlorine and iron peaks confirming the chemical nature of the layers.

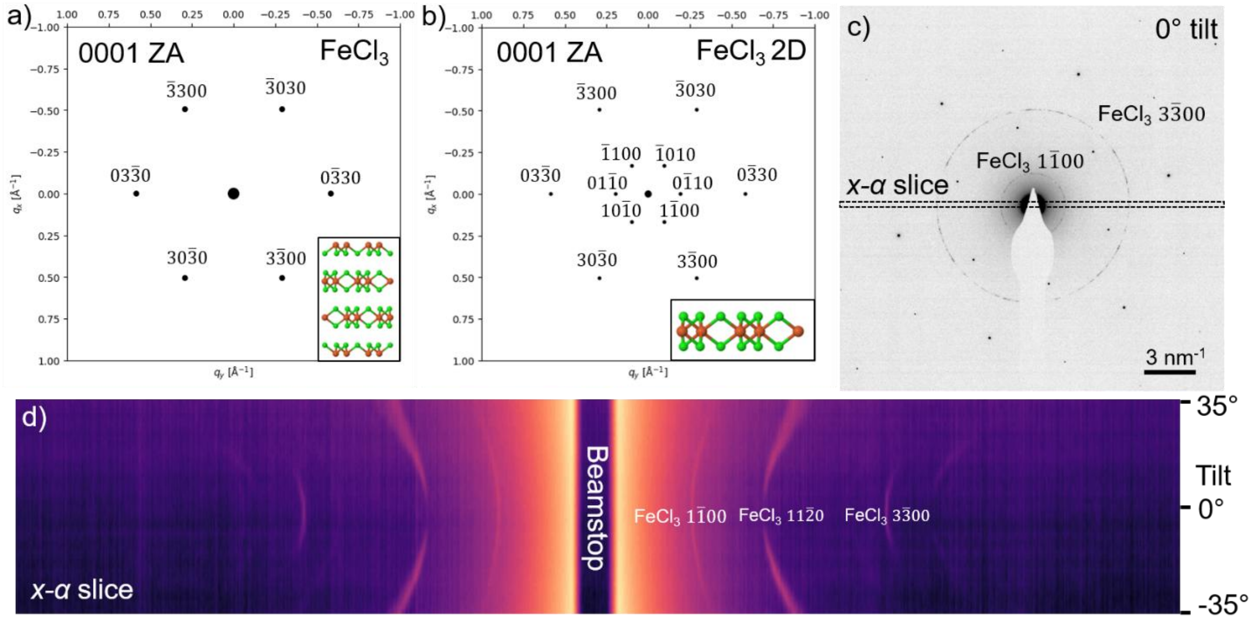

Supplementary Figure 5: Simulated diffraction patterns and diffraction tilting. a) Simulated electron diffraction pattern for $FeCl_3$ in 0001 zone axis orientation. The first allowed set of reflections are the $3\bar{3}00$ type reflections. b) Simulated diffraction pattern for 2D $FeCl_3$, showing the appearance of $1\bar{1}00$ type reflections. c) Experimental SAED pattern of intercalated graphite, showing graphite reflections and $FeCl_3$ rings. This pattern is part of a diffraction tilt series (shown in supplementary movie 1). The occurrence of the $1\bar{1}00$ ring confirms the quasi 2D nature of the intercalated layers. d) *x-α* slice through the diffraction tilt series showing the position and relative intensity of $FeCl_3$ reflections in dependence of the tilt angle. The $1\bar{1}00$ reflection stays visible for all tilt angles, but the apparent distance changes. This behavior is typical for 2D materials and can be explained by the different positions at which the Ewald's sphere cuts the reciprocal lattice rods of $FeCl_3$. While the $11\bar{2}0$ reflection is not visible at 0° tilt it becomes visible at all non-zero tilt angles and also changes apparent spacing with tilt angle.

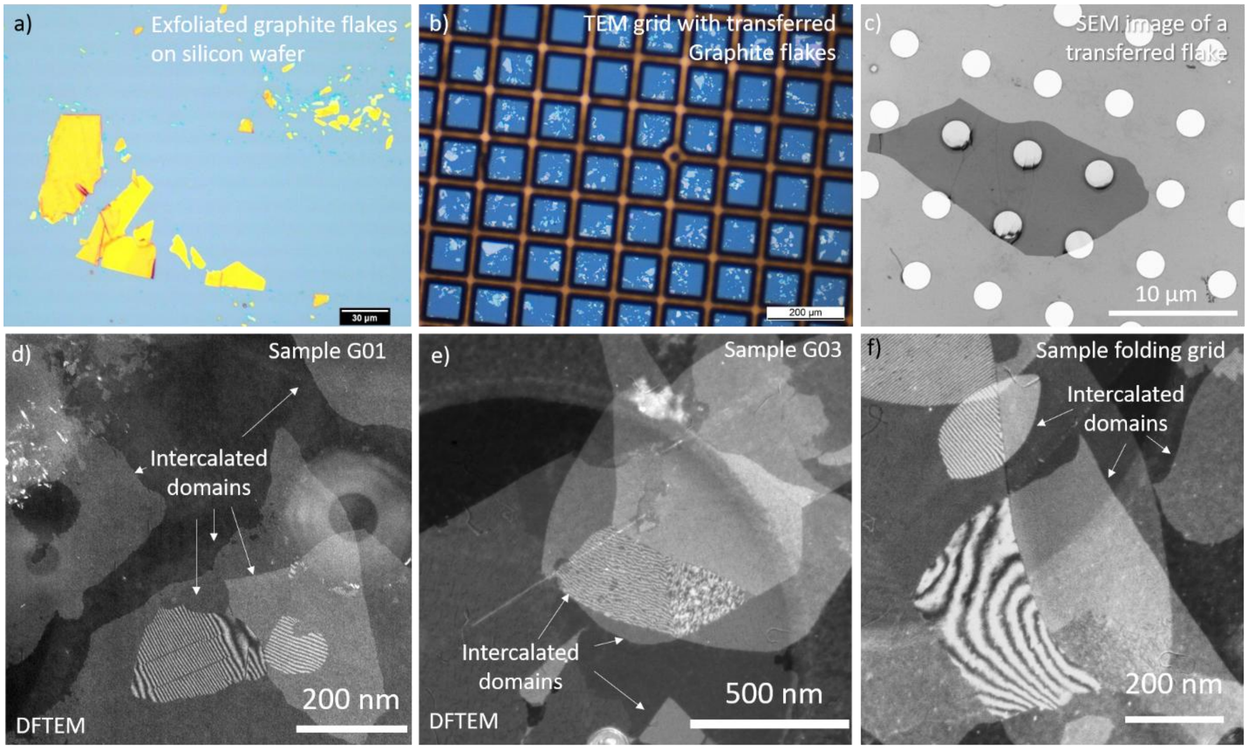

Supplementary Figure 6: Sample preparation and reproducibility of intercalation. a)-c) different steps in the preparation procedure starting from mechanical exfoliation of graphite and transfer to silicon wafers. This is then followed by a transfer step to TEM grids in which a lot of electron beam transparent flakes are transferred. d)-f) different samples showing the same principal intercalation structure. Notably the sample shown in f) is a bulk-like graphite piece in a folding grid. Overlapping domains lead to the formation of moiré fringes which are highly sensitive to strain and defects (see for example the change in fringe spacing and defect lines in the bottom layer in d).

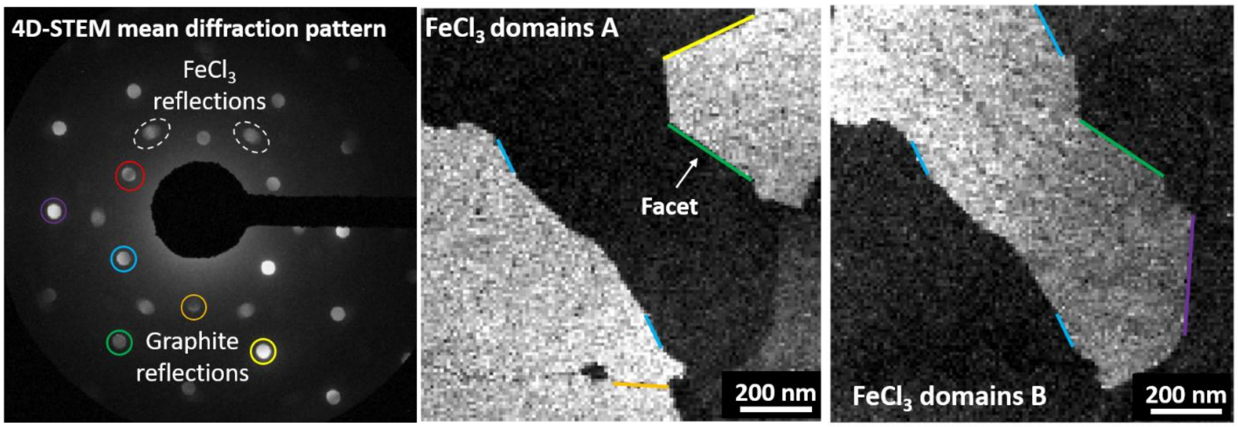

Supplementary Figure 7: 4D-STEM scan of $FeCl_3$ in graphite, containing several intercalated layers. The layers are rotated approximately 30 ° with respect to the graphite lattice. There are some straight facets visible at the edges of the intercalated layers which line up with graphite lattice planes. Other segments of the outline do not line up with graphite lattice planes, indicating a weak correlation of the two.

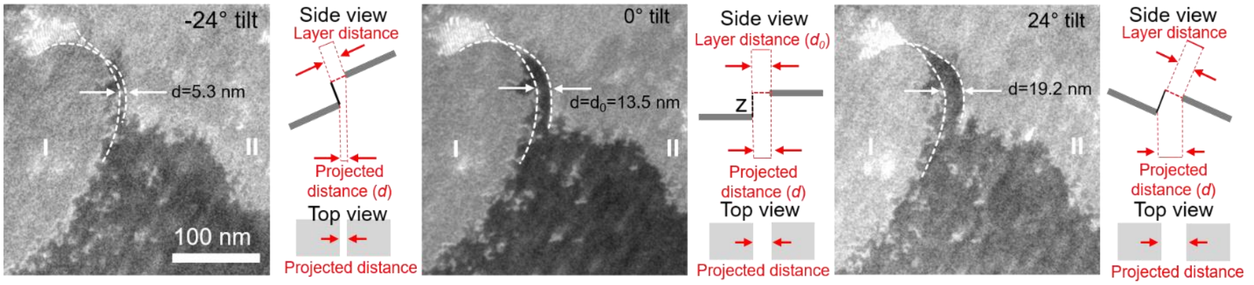

Supplementary Figure 8: Tilting experiment and reconstruction of 3D structure of the layers. At 0° tilt, we can measure the initial lateral distance (or overlap) between two layers (d_0) since this condition imaging the projection normal to the graphite flake. During tilting the projected distance/overlap between the layers (d) changes depending on the vertical distance (z) between them. with the initial distance, the projected distance and the tilt angle (α), the vertical separation between the layers can be calculated as follows: $z = \frac{d - d_0 \cos\alpha}{\sin\alpha}$. In this example the initial distance between the layers is 13.5 nm (at the position marked by arrows). After tilting to -24° the projected overlap is only 5.3 nm. This leads to a calculated z-spacing of around 17 nm. Tilting in the other direction leads to a projected distance of 19.2 nm which gives us the same value. By segmenting the lateral extent of the domains at 0° tilt and the vertical separation, the full 3D arrangement of the layers can be calculated. The thickness of the graphite flake is estimated by the relative movement of surface contamination during tilting.

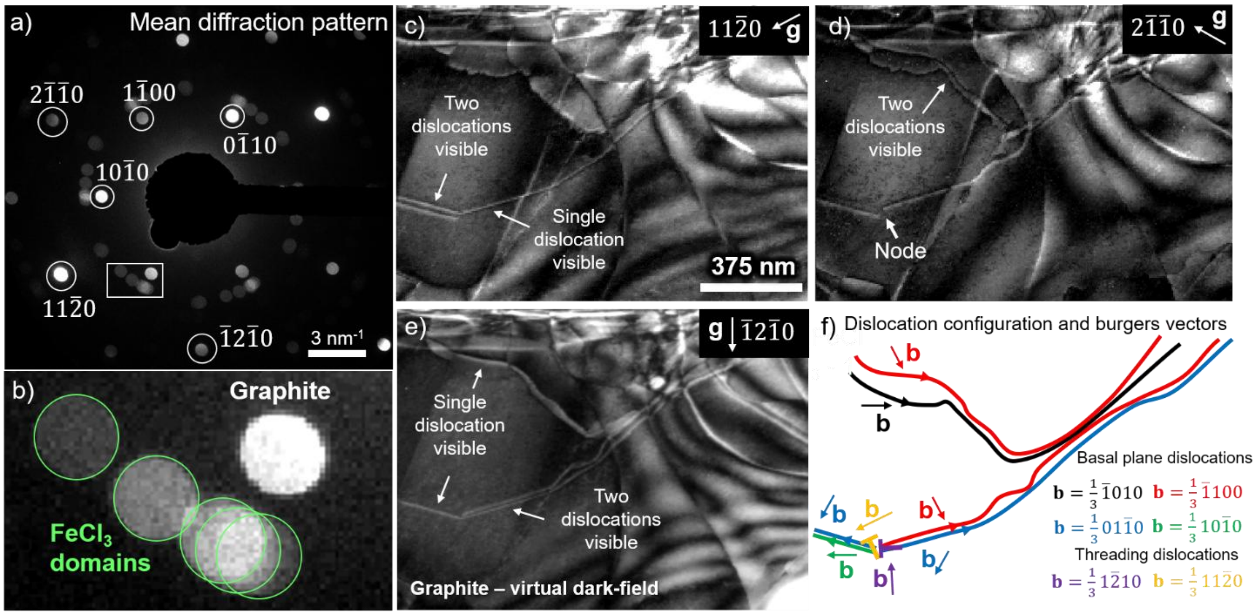

Supplementary Figure 9: 4D-STEM based burgers vector analysis of graphite dislocations. a) Mean diffraction pattern of the 4D-STEM scan shown in Figure 3. b) Closeup-view of overlapping discs originating from rotationally misaligned $FeCl_3$. The combined signal from all these discs is used for the reconstruction of intercalated domains in Figure 3. c)-e) virtual dark-field images based on $11\bar{2}0$ type graphite reflections as highlighted in a). In each dark-field image different basal plane dislocations are visible which can be used to identify their burgers vector in accordance with the g.b=0 invisibility criterion. As the dislocations are basal plane dislocations this restricts the burgers vector to be of $1\bar{1}00$ type. Upon closer inspection

there is a dislocation node present in the lower left of the graphite flake. This node indicates that there must be at least one more dislocation present that is not contained in the basal plane so that the sum of burgers vectors at the intersection is zero. Such out of plane (perfect) dislocations have been observed previously. Using the sum rule of burgers vectors, the burgers vector of these dislocations can be inferred as well. The line sense used is indicated by small triangles. We must note that the sign of the burgers vectors has been arbitrarily chosen for one dislocation and then propagated to the rest of the defects accordingly. The entire dislocation structure is shown in f) with the burgers vectors of all defects. Dislocation line segments that are parallel to the burgers vector are of screw type and conversely if they are perpendicular the line segment is of edge type.

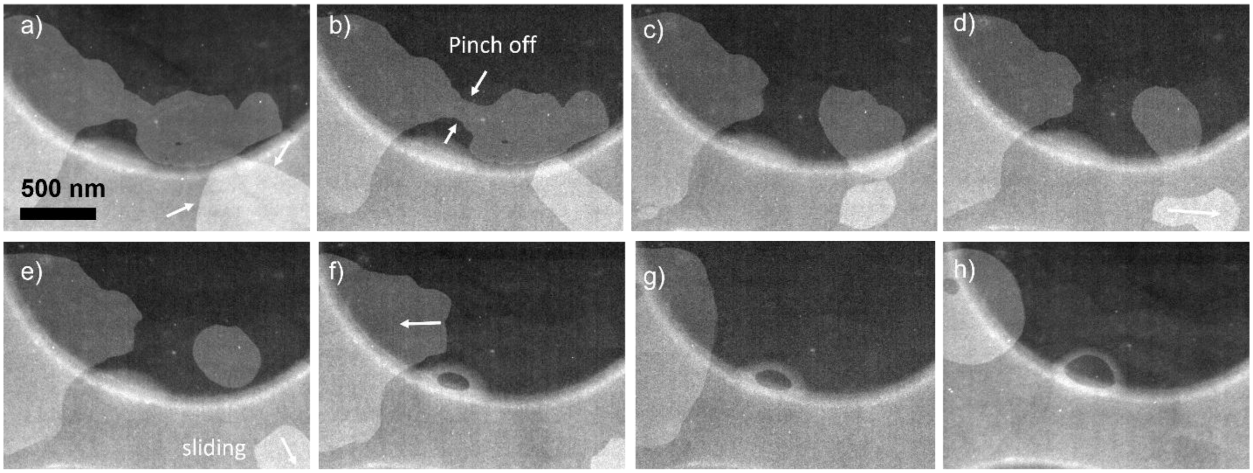

Supplementary Figure 10: *In situ* heating and dissolution of domains. During heating (up to 350 °C), $FeCl_3$ domains start to shrink and change their shape. Continuous domains can be split in two (see b & c) and some domains start to slide while keeping their overall area (see d to f). Some domains remain after the heating process (h) which may be explained by the increasing vapor pressure of $FeCl_3$ that remains in the interlayer gaps of graphite. Full movie in supplementary movie 3.

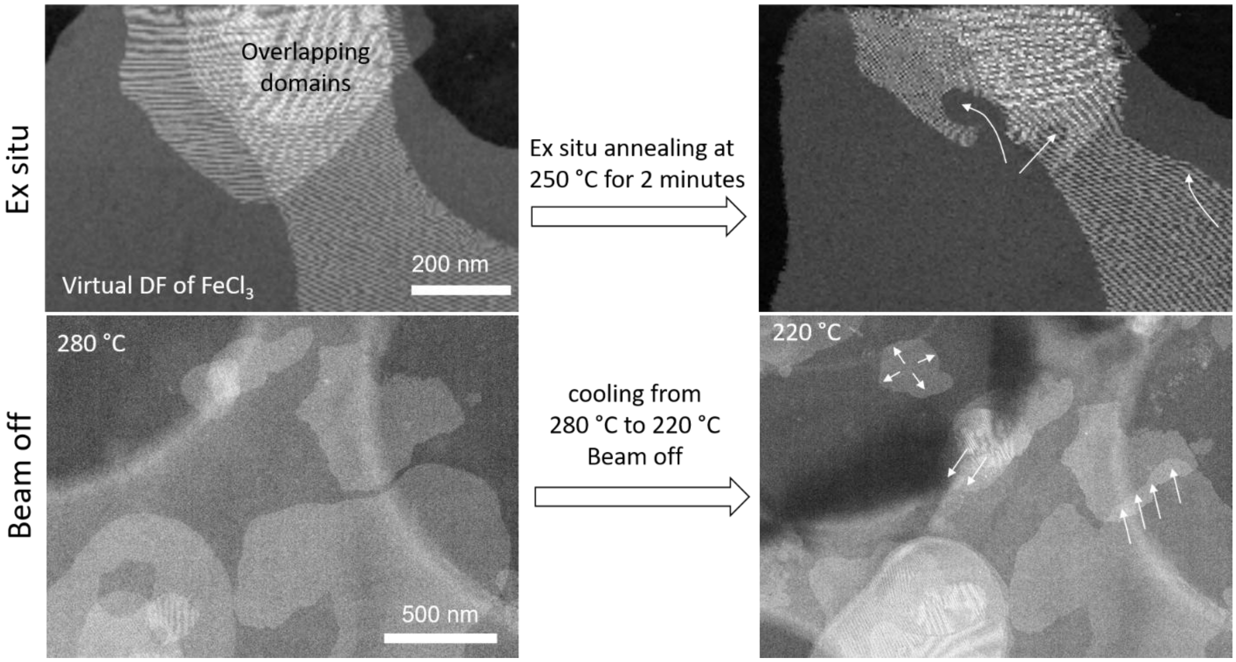

Supplementary Figure 11: Control experiments to see whether the observed transformations during in situ heating are thermally driven. Top: Ex situ heating experiment in which a 4D-STEM scan of a sample area was performed and then the sample was put on a hot plate at 250 °C for 2 minutes. After that another 4D-STEM scan of the same area was performed. Even though the heating cycle was short, a significant change of the domain structure can be observed. Bottom: Beam-off experiment in which the beam was switched off during cooling from 280 to 220 °C. Comparing the images before and after cooling reveals growth of existing domains and the appearance of a new intercalated domain.

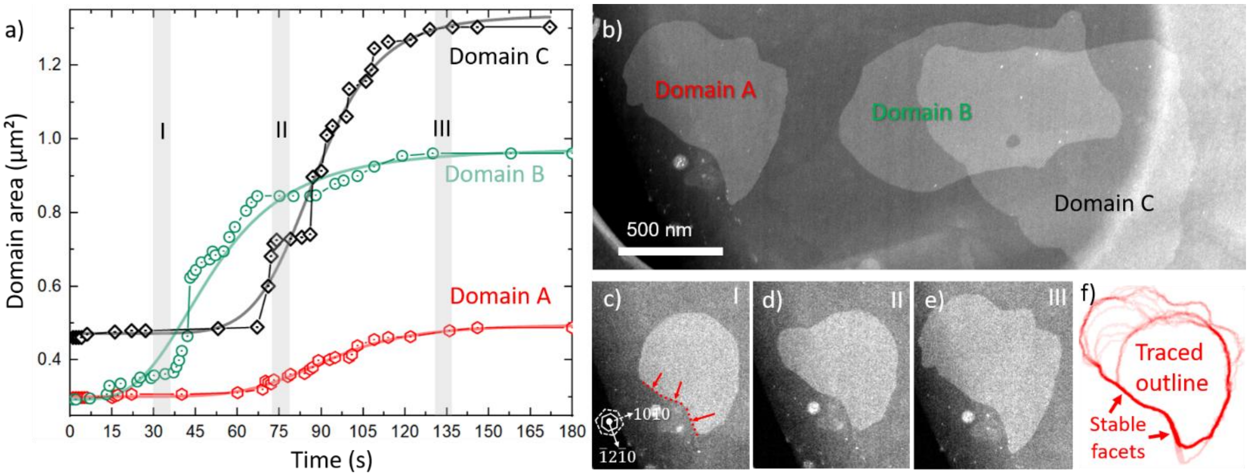

Supplementary Figure 12: Quantitative analysis of domain growth during in situ heating and cooling. a) Area over time graph of three domains that have been tracked showing a growth behavior following a logistics curve. b) Image after growth, showing the final domains. c)-e) exemplary images of Domain A during domain growth. Some facets appear that follow certain planes of the Graphite host lattice. These

facets are stable during growth as shown by the overlaid outlines of the domain over time (f). Source data are provided as Source Data file.

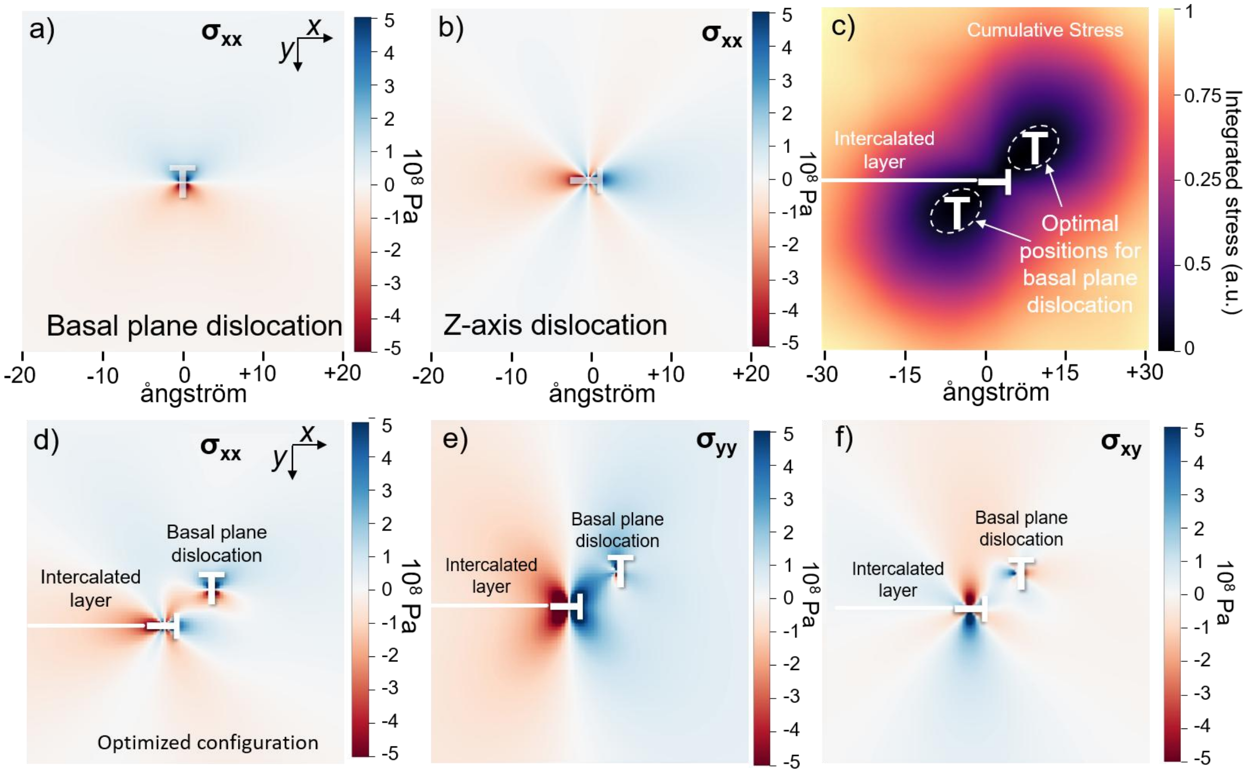

Supplementary Figure 13: Stress field interaction of intercalated layer and basal plane dislocation. a) Calculated stress field of a basal plane edge dislocation in graphite in *x* direction. b) Calculated stress field of z-axis edge dislocation (intercalated layer edge) in graphite in *x* direction. c) Overlap of stress fields from basal plane dislocation and intercalated layer edge for varying positions. The layer edge as fixed and the position of the basal plane dislocation is varied. The color scale represents the total integrated stress for a specific position. There are two minima in close vicinity to the fixed dislocation, which means that there is an attraction between the two defects. Strain field for the optimum dislocation configuration in *xx* (d), *yy* (e) and *xy* (f).

The stress has been calculated using the following set off equations (taken from "Introduction to Dislocations" by Hull & Bacon). We assumed simple elasticity theory and treated the edge of the intercalated layer as a z-axis dislocation in graphite.

$$\sigma_{xx} = -\frac{Gb}{2\pi(1-\nu)} \cdot y \frac{3x^2 + y^2}{(y^2 + y^2)^2}$$

$$\sigma_{yy} = \frac{Gb}{2\pi(1-\nu)} \cdot y \frac{x^2 - y^2}{(y^2 + y^2)^2}$$

$$\sigma_{xy} = \frac{Gb}{2\pi(1-\nu)} \cdot x \frac{x^2 - y^2}{(y^2 + y^2)^2}$$

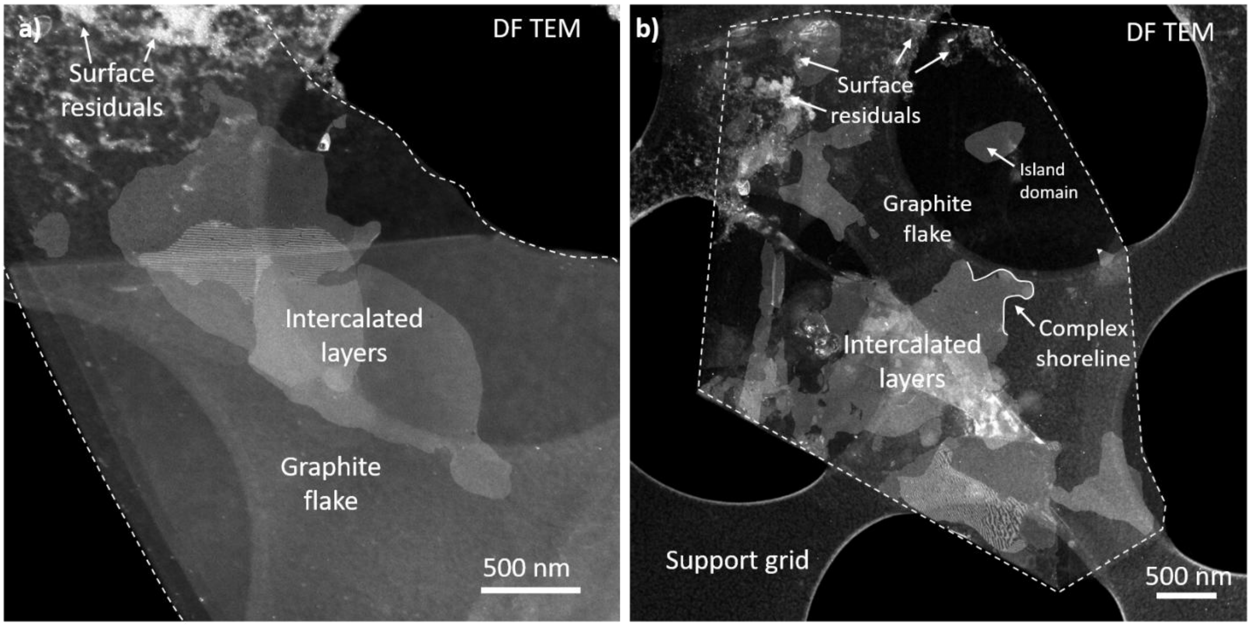

Supplementary Figure 14: DF-TEM images of samples without the final washing step. The samples show the same type of intercalated structures with complex shorelines and isolated domains as the washed samples. In the unwashed samples, residuals from the synthesis are deposited on the specimen surface, which obscures the view on some of the intercalated layers.
