# Supplementary material for: Staging and defect-limited intercalation of FeCl3 in graphite electrodes
Source: Nat Commun. 2026 Jun 16;17:7609. doi: 10.1038/s41467-026-74399-w (PMC13424089; doi:10.1038/s41467-026-74399-w)
Supplement: Supplementary file 2 — Description of Additional Supplementary Files [file 41467_2026_74399_MOESM2_ESM.pdf]

Description of Additional Supplementary Files

File Name: Supplementary Movie 1
Description: Diffraction tilt series. Selected area electron diffraction patterns acquired from intercalated graphite at different angles from 0 ° to 35 ° maximum tilt. Discrete spots can be attributed to single crystalline graphite, whereas FeCl3 produces complete rings in the diffraction pattern. During tilting the rings become elliptical because the Ewald's sphere cuts the reciprocal lattice rods of the 2D structure at different positions. The increase in projected thickness during tilting leads to an increase in the background signal in the diffraction patterns.

File Name: Supplementary Movie 2
Description: Dark-field tilt series of intercalated layers. The tilt series runs from -24 to +24 degrees. The objective aperture was placed approximately in line with the tilt axis to keep the intercalated layers visible throughout tilting. The aperture position needed to be slightly readjusted during tilting to keep all the domains visible which was not perfectly done for all tilt angles.

File Name: Supplementary Movie 3
Description: Domain dissolution during in situ heating. Upon heating the intercalation compound to 350 °C, FeCl3 domains start to shrink and dissolve. One of the domains is split into two parts before further dissolution. Domain sliding is observed in the lower part of the video. The dissolved FeCl3 species most likely freely diffuse within the van-der-Waals gap of graphite.

File Name: Supplementary Movie 4
Description: Domain growth observed during in situ cooling from 350 °C to 220 °C. The excess $FeCl_3$ contained in the graphite van-der-Waals gaps (due to prior heating and dissolution of domains) starts to recrystallize over time which leads to the growth of pre-existing intercalated domains. The growth proceeds in steps of high growth rates followed by inactivity. Domain growth does occur on overlapping layers at the same time.

File Name: Supplementary Movie 5
Description: In situ cooling from 350 °C to 220 °C at a location where dislocations are present. The shape of the growing domains is strongly influenced by the defects present in the host lattice.
