# Supplementary figures and images for: Staging and defect-limited intercalation of FeCl3 in graphite electrodes
Source: Nat Commun. 2026 Jun 16;17:7609. doi: 10.1038/s41467-026-74399-w (PMC13424089; doi:10.1038/s41467-026-74399-w)

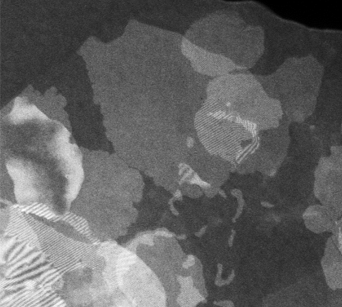

Supplement: Supplementary file 9 — Source Data [file 41467_2026_74399_MOESM9_ESM.zip › Figure1_b.tif]

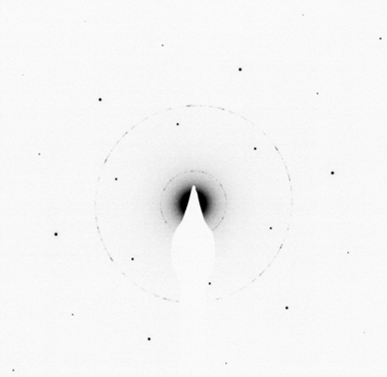

Supplement: Supplementary file 9 — Source Data [file 41467_2026_74399_MOESM9_ESM.zip › Figure1_c_A.tif]

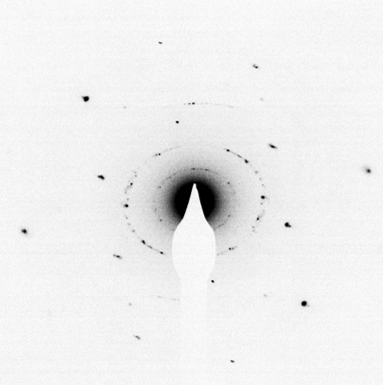

Supplement: Supplementary file 9 — Source Data [file 41467_2026_74399_MOESM9_ESM.zip › Figure1_c_B.tif]

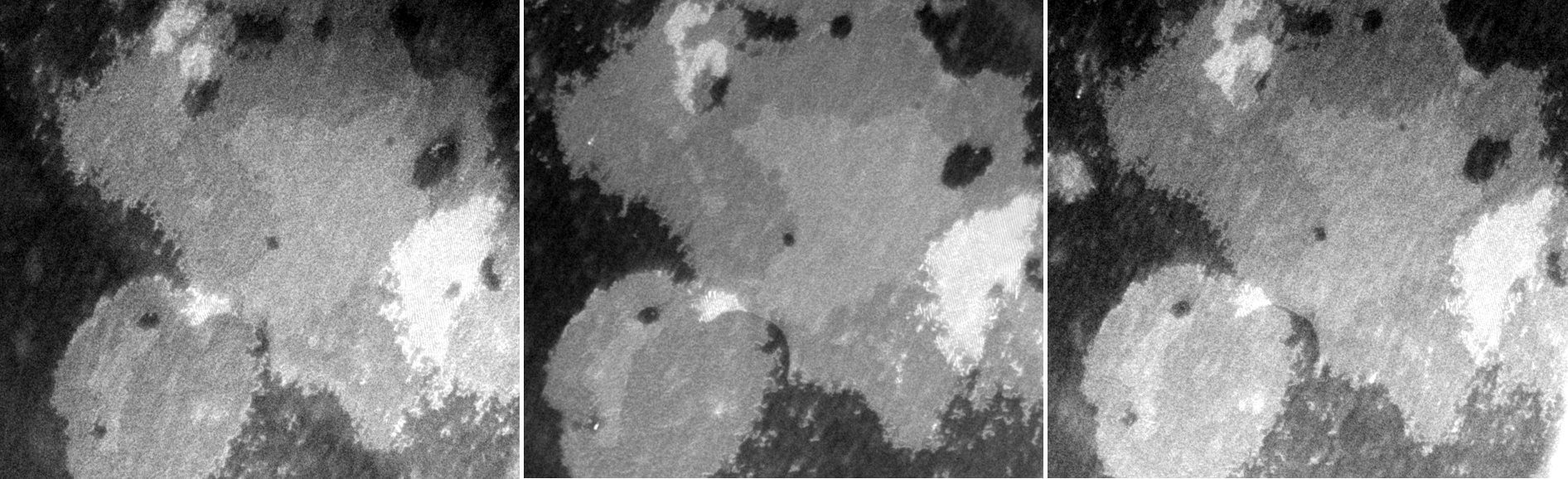

Supplement: Supplementary file 9 — Source Data [file 41467_2026_74399_MOESM9_ESM.zip › Figure2.tif]

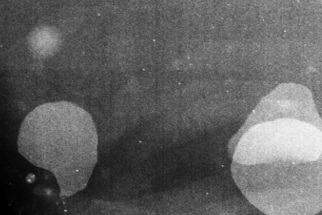

Supplement: Supplementary file 9 — Source Data [file 41467_2026_74399_MOESM9_ESM.zip › Figure4_a.tif]

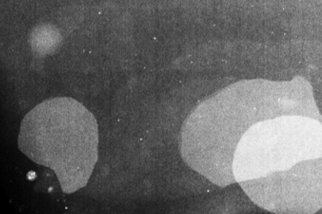

Supplement: Supplementary file 9 — Source Data [file 41467_2026_74399_MOESM9_ESM.zip › Figure4_b.tif]

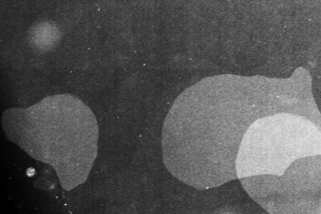

Supplement: Supplementary file 9 — Source Data [file 41467_2026_74399_MOESM9_ESM.zip › Figure4_c.tif]

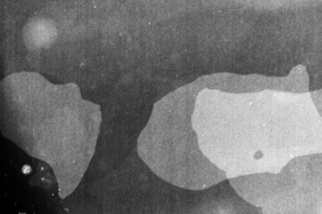

Supplement: Supplementary file 9 — Source Data [file 41467_2026_74399_MOESM9_ESM.zip › Figure4_d.tif]

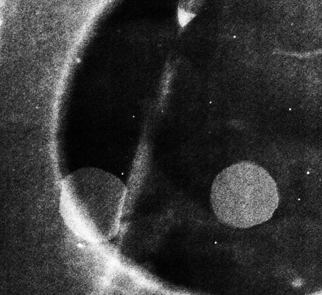

Supplement: Supplementary file 9 — Source Data [file 41467_2026_74399_MOESM9_ESM.zip › Figure4_e.tif]

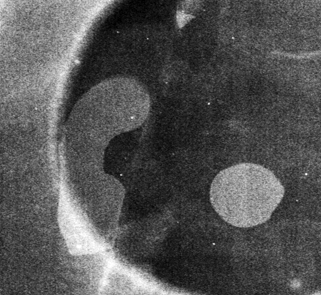

Supplement: Supplementary file 9 — Source Data [file 41467_2026_74399_MOESM9_ESM.zip › Figure4_f.tif]

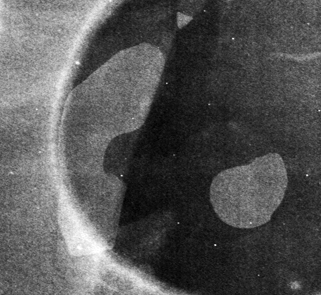

Supplement: Supplementary file 9 — Source Data [file 41467_2026_74399_MOESM9_ESM.zip › Figure4_g.tif]

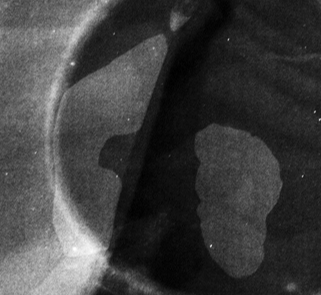

Supplement: Supplementary file 9 — Source Data [file 41467_2026_74399_MOESM9_ESM.zip › Figure4_h.tif]

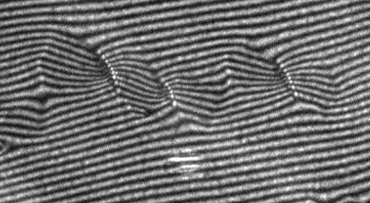

Supplement: Supplementary file 9 — Source Data [file 41467_2026_74399_MOESM9_ESM.zip › Figure4_i.tif]

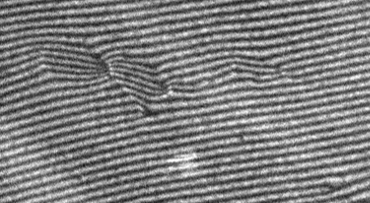

Supplement: Supplementary file 9 — Source Data [file 41467_2026_74399_MOESM9_ESM.zip › Figure4_j.tif]

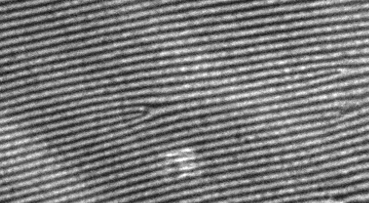

Supplement: Supplementary file 9 — Source Data [file 41467_2026_74399_MOESM9_ESM.zip › Figure4_k.tif]
