# Supplementary material for: Staging and defect-limited intercalation of FeCl3 in graphite electrodes
Source: Nat Commun. 2026 Jun 16;17:7609. doi: 10.1038/s41467-026-74399-w (PMC13424089; doi:10.1038/s41467-026-74399-w)
Supplement: Supplementary file 8 — Transparent Peer Review file [file 41467_2026_74399_MOESM8_ESM.pdf]

nature portfolio

Peer Review File

Staging and Defect Limited Intercalation of FeCl3 in Graphite Electrodes

Corresponding Author: Professor Andrew Minor

This manuscript has been previously reviewed at another journal. This document only contains information relating to versions considered at Nature Communications.

This file contains all reviewer reports in order by version, followed by all author rebuttals in order by version.

Version 0:

Reviewer comments:

Reviewer #1

(Remarks to the Author)
I have examined the newly added EDS data. The spectra clearly confirm the presence of both Fe and Cl signals. However, in the 2D elemental maps, only the distributions of C and Cl are presented in the color overlay, which supports that the intercalated domains contain Cl. It is not clear why the Fe signal was not also included in the color map. Showing the Fe distribution would provide more direct and complete compositional confirmation of the intercalated phase.

Overall, I am reasonably confident that the intercalated material is FeCl3. In my view, the central contribution of this work lies in the methodology used to visualize intercalation migration and to elucidate how edge defects in graphite constrain the motion of the intercalants. Whether the intercalated species is strictly FeCl2, FeCl3, or a partially oxidized form is not the most critical aspect of the manuscript. Nevertheless, clearer compositional confirmation, particularly through explicit Fe mapping, would strengthen the paper and remove any remaining ambiguity.

Reviewer #2

(Remarks to the Author)
The manuscript entitled “Staging and Defect Limited Intercalation of $FeCl_3$ in Graphite Electrodes” discusses the spatial distribution and growth process of $FeCl_3$ intercalation within graphite interlayers as revealed by TEM.

Through the previous rounds of review, the technical issues that needed to be pointed out have, in my view, been resolved.

On the other hand, I agree with the concern about overclaiming raised by Reviewer 1 comment 3 and 4, as shown below. However, Ithe revised version I reviewed still does not sufficiently reflect these concerns.

Regarding point 3, the authors should more clearly distinguish between the aspects of the TEM observations that support previously proposed mechanisms and the phenomena newly identified here that were not anticipated in earlier models, and they should formulate their conclusions accordingly.

Regarding point 4, for example, the authors should explicitly state both the possibility that the present model may be partially applicable to Li intercalation and the possibility that it may not be fully applicable. I agree that this paper is highly interesting and important. However, I believe that a more critical delimitation of the scope of applicability of the findings would be useful for eaders.

3. Defect Interaction: The rebuttal argues that commensurate intercalants like Li would be "even more strongly affected" by dislocations. While plausible, this remains speculative. The "island" formation mechanism for a large, rotationally disordered molecule like FeCl3 cannot be confidently extrapolated to Li-intercalation dynamics without direct comparative data.

4. The authors present the "Stochastic Simultaneous Intercalation Model" (Figure 5) as a key outcome. However, this model appears to be an incremental refinement rather than a conceptual leap. Island Growth: The existence of island domains

(Daumas-Hérold domains) is the established consensus for GIC phase transitions, as opposed to the Rüdorff-Hoffmann model. Defect Nucleation: The observation that nucleation occurs at defects rather than strictly at edges is a manifestation of heterogeneous nucleation, a fundamental concept in materials science. Incremental Advance: While the 4D-STEM visualization is high-quality, the conceptual model confirming that "defects limit the extent of intercalation" confirms existing theory rather than challenging it or establishing a new paradigm.

Version 1:

Reviewer comments:

Reviewer #2

(Remarks to the Author)
In the revised version, the authors have addressed my comments appropriately. Therefore, I recommend acceptance.

Open Access This Peer Review File is licensed under a Creative Commons Attribution 4.0 International License, which permits use, sharing, adaptation, distribution and reproduction in any medium or format, as long as you give appropriate credit to the original author(s) and the source, provide a link to the Creative Commons license, and indicate if changes were made.
In cases where reviewers are anonymous, credit should be given to 'Anonymous Referee' and the source.
The images or other third party material in this Peer Review File are included in the article's Creative Commons license, unless indicated otherwise in a credit line to the material. If material is not included in the article's Creative Commons license and your intended use is not permitted by statutory regulation or exceeds the permitted use, you will need to obtain permission directly from the copyright holder.
To view a copy of this license, visit https://creativecommons.org/licenses/by/4.0/

Response to the reviewers

We thank the reviewers for once again evaluating our manuscript. A response to their points is attached below. Changes made to the manuscript and supplementary information as a result of this revision are marked in green.

Reviewer #1

I have examined the newly added EDS data. The spectra clearly confirm the presence of both Fe and Cl signals. However, in the 2D elemental maps, only the distributions of C and Cl are presented in the color overlay, which supports that the intercalated domains contain Cl. It is not clear why the Fe signal was not also included in the color map. Showing the Fe distribution would provide more direct and complete compositional confirmation of the intercalated phase. Overall, I am reasonably confident that the intercalated material is FeCl3. In my view, the central contribution of this work lies in the methodology used to visualize intercalation migration and to elucidate how edge defects in graphite constrain the motion of the intercalants. Whether the intercalated species is strictly FeCl2, FeCl3, or a partially oxidized form is not the most critical aspect of the manuscript. Nevertheless, clearer compositional confirmation, particularly through explicit Fe mapping, would strengthen the paper and remove any remaining ambiguity.

We thank the reviewer for suggesting to add the Fe Signal. In the first iteration we left the signal out for clarity reasons since the counts were lower compared to the Cl signal. However, we agree that adding the extra information is beneficial. In response to this point we added the Fe elemental map in supplementary figure 3 and now show C, Fe and Cl colormaps separately.

Reviewer #2 (Remarks to the Author):

We thank reviewer #2 for taking up the task to re-evaluate our manuscript. A response to their points follows bellow.

The manuscript entitled "Staging and Defect Limited Intercalation of $FeCl_3$ in Graphite Electrodes" discusses the spatial distribution and growth process of $FeCl_3$ intercalation within graphite interlayers as revealed by TEM. Through the previous rounds of review, the technical issues that needed to be pointed out have, in my view, been resolved.

We thank the reviewer for this assessment of the improvements made in the reviewing process.

On the other hand, I agree with the concern about overclaiming raised by Reviewer 1 comment 3 and 4, as shown below. However, the revised version I reviewed still does not sufficiently reflect these concerns. Regarding point 3, the authors should more clearly distinguish between the aspects of the TEM observations that support previously proposed mechanisms and the phenomena newly identified here that were not anticipated in earlier models, and they should formulate their conclusions accordingly.

We thank the reviewer for suggesting to more clearly formulate our observations and conclusion in regards to previously proposed phenomena and mechanisms. In response to this point we rephrased parts of the discussion section.

Regarding point 4, for example, the authors should explicitly state both the possibility that the present model may be partially applicable to Li intercalation and the possibility that it may not be fully applicable. I agree that this paper is highly interesting and important. However, I believe that a more critical delimitation of the scope of applicability of the findings would be useful for readers.

We thank the reader again for suggesting to clarify our statements. In response we now clearly qualify the applicability of our findings in regard to lithium and clearly state that they may not be transferrable.
